# Variability in Biomarkers of Arsenic Exposure and Metabolism in Adults over Time

**DOI:** 10.1289/ehp.11251

**Published:** 2008-11-19

**Authors:** Molly L. Kile, Elaine Hoffman, Yu-Mei Hsueh, Sakila Afroz, Quazi Quamruzzaman, Mahmuder Rahman, Golam Mahiuddin, Louise Ryan, David C. Christiani

**Affiliations:** 1 Harvard School of Public Health, Boston, Massachusetts, USA;; 2 Department of Public Health, School of Medicine, Taipei Medical University, Taipei, Taiwan, Republic of China;; 3 Dhaka Community Hospital, Dhaka, Bangladesh

**Keywords:** arsenic, arsenic metabolism, Bangladesh, biomarkers, exposure assessment, intraclass correlation, urinary arsenic metabolites

## Abstract

**Background:**

Urinary arsenic metabolites (UAs) are used as biomarkers of exposure and metabolism.

**Objectives:**

To characterize inter- and intraindividual variability in UAs in healthy individuals.

**Methods:**

In a longitudinal study conducted in Bangladesh, we collected water and spot urine samples from 196 participants every 3 months for 2 years. Water arsenic (As) was measured by inductively coupled plasma–mass spectrometry and urinary As [arsenite, arsenate, monomethylarsonic acid (MMA), and dimethylarsinic acid (DMA)] were detected using high-performance liquid chromatography–hydride-generated atomic absorption spectrometry. We used linear mixed-effects models to compute variance components and evaluate the association between UAs and selected factors.

**Results:**

The concentrations of UAs were fairly reproducible within individuals, with intraclass correlation coefficients (ICCs) of 0.41, 0.35, 0.47, and 0.49 for inorganic As (InAs), MMA, DMA, and total urinary As (TUA). However, when expressed as a ratio, the percent InAs (%InAs), %MMA, and %DMA were poorly reproducible within individuals, with ICCs of 0.16, 0.16, and 0.17, respectively. Arsenic metabolism was significantly associated with sex, exposure, age, smoking, chewing betel nut, urinary creatinine, and season. Specificity and sensitivity analyses showed that a single urine sample adequately classified a participant’s urinary As profile as high or low, but TUA had only moderate specificity for correctly classifying drinking water exposures.

**Conclusions:**

Epidemiologic studies should use both urinary As concentrations and the relative proportion of UAs to minimize measurement error and to facilitate interpretation of factors that influence As metabolism.

Chronic exposure to arsenic (As) contaminated drinking water is an international environmental health problem (World Health Organization 1999). Once ingested, inorganic As (InAs) is metabolized through a series of reduction and oxidative methylation reactions to form monomethylarsonic acid (MMA) and dimethylarsinic acid (DMA) (Kitchin 2001). Human ingestion experiments performed by Buchet et al. (1981) indicated that As biotransformation follows first-order rate constants and that urinary As metabolites (UAs) have a half-life ranging from 39 to 59 hr. When individuals are at steady state, approximately 60% of the total ingested dose is excreted in the urine daily. Despite its short half-life, total urinary As (TUA) is commonly used as a biomarker of exposure and is positively correlated with As concentrations in drinking water in chronically exposed populations (Calderon et al. 1999; Hopenhayn-Rich et al. 1996).

In addition to TUA, the percentage of each urinary As species is used as a biomarker of As metabolism. Population-based studies reveal considerable interindividual variability in urinary As levels, with urine containing 10–30% InAs, 10–20% MMA, and 60–70% DMA (Calderon et al. 1999; Hopenhayn-Rich et al. 1996). Understanding the factors that contribute to this observed interindividual variability in UAs is of growing interest because epidemiologic studies suggest that an individual’s ability to metabolize InAs is a risk factor for As-related toxicity. For instance, studies in Mexico, Taiwan, and Bangladesh have shown that individuals who have a higher proportion of InAs and MMA and lower DMA in urine have an increased risk of As-induced skin lesions (McCarty et al. 2007), skin cancer (Chen et al. 2003a; Hsueh et al. 1997; Yu et al. 2000), and bladder cancer (Chen et al. 2003b; Steinmaus et al. 2006).

However, diseases associated with chronic As exposure have long latency periods, and it is unclear how stable UAs are within an individual over a long time period. To date, only two studies have examined intraindividual variability in UAs. Concha et al. (2002) analyzed UAs from 15 women chronically exposed to As contaminated drinking water in Chile and observed no significant daily intraindividual variability in the relative proportion or concentration of UAs over a 5-day period. Steinmaus et al. (2005) examined intraindividual variability in UAs in 81 individuals with a history of moderate to high As exposures who participated in a case–control study of bladder cancer in the United States and found that the relative proportions of UAs were fairly stable within individuals over an average interval of 258 days. However, the observation periods for these studies were relatively short given the long latency for As-related diseases such as cancer. Also, Steinmaus et al. (2005) included participants with bladder cancer, who may have altered As methylation capacity.

Therefore, we conducted a 4-year prospective repeated-measures biomonitoring study in Bangladesh to evaluate inter- and intraindividual variability in UAs over a long time period. We recruited individuals residing in an As endemic region of Bangladesh who do not exhibit any dermal symptom of As toxicity. Our primary aim was to examine inter- and intraindividual sources of variability in UAs expressed as concentrations and as proportions of the TUA in an adult population. A secondary aim was to conduct sensitivity and specificity analyses to determine how well a single urine sample predicted an individual’s urinary As profile and As exposure. This analysis presents the first 2 years of urinary As biomonitoring measurements, reflecting currently available data.

## Materials and Methods

### Study design and participant selection

We recruited participants through a series of community meetings held in Pabna, Bangladesh. Individuals were eligible for this study if they were long-term residents of Pabna, obtained their drinking water from a private tube well, and received primary health care from the Pabna Community Clinic, an affiliate of Dhaka Community Hospital, and if several members within each household were willing to participate. The primary rationale for multiple persons per household was to facilitate sample collection in rural areas.

During the initial visit in September 2001, a behavioral and demographic questionnaire was administered and blood, urine, toenail, and water samples were collected. Researchers then visited participants at their homes every 3 months for 4 years to collect urine, toenail, and water samples. In this analysis, we used data collected from April 2002 through March 2004, representing eight sampling collection periods and reflecting currently available data. Beginning in the fourth sampling period, urine and drinking water was collected from each participant for 3 consecutive days during each sampling period to capture potential short-term variability.

Overall, we enrolled 50 households (*n* = 248 participants) in this study. Residents in two households (*n* = 13) moved out of the study area before April 2002 to seek employment in Dhaka. Residents from one household (*n* = 3) were diagnosed with As-induced skin lesions; we did not include them in this analysis because of the possibility that individuals exhibiting symptoms of chronic As toxicity may have altered As metabolism, and we wanted to examine As methylation in a more generalizable population. Of the remaining participants, we excluded 29 children younger than 15 years because As metabolism may be different in children compared with adults (Concha et al. 1998). Another four subjects diagnosed with diabetes were also excluded because they may have altered kidney function that could influence As excretion. Subsequently, in this analysis we used data from 195 participants residing in 47 households.

The institutional review boards at Harvard School of Public Health and Dhaka Community Hospital approved the protocol for this study. Informed consent was obtainedfrom all adult participants before participation and parental consent was obtained for all participants younger than 18 years.

### Water sample collection and analysis

We collected drinking water samples from the tube well identified by the household members as their primary source of drinking water. Tube wells were purged by pumping the well for several minutes before 50 mL of water was collected in an acid-washed polypropylene tube (BD Falcon, BD Bioscience, Bedford, MA, USA). Samples were preserved with reagent-grade HNO_3_ (Merck, Damstadt, Germany) to pH < 2, shipped to the Harvard laboratory, and kept at room temperature until analysis. We quantified total InAs by inductively coupled plasma–mass spectrometry using U.S. Environmental Protection Agency method 200.8 (Environmental Laboratory Services, North Syracuse, NY, USA). Analysis was validated using PlasmaCAL multielement QC standard #1 solution (SCP Science, Baíe Dúrfé, Quebec, Canada). The average percent recovery for InAs was 96.0 ± 2.9%. The limit of detection (LOD) for this method was 1 μg As/L. We assigned samples below the LOD a value of 0.5 μg As/L.

### Urine sample collection and analysis

Participants were visited in their homes the day before urine samples were scheduled to be collected, provided with sterile urine collection containers (VWR International, West Chester, PA, USA), and instructed to collect a first-void urine sample. Technicians collected the urine samples in the morning, placed them on ice, and transported them to Pabna Community Clinic, where they were transferred into 15mL polyethylene tubes (BD Falcon) and immediately frozen at −20°C. All samples were processed within several hours of collection. Samples were then shipped on dry ice to Dhaka, re-packaged with more dry ice, and then shipped overnight to the environmental chemistry laboratory of Taipei Medical University for analysis.

Frozen urine samples were thawed at room temperature, dispersed by ultrasonic waves, and filtered through a Sep-Pak C18 column to remove protein (Mallinckrodt Baker Inc., Phillipsburg, NJ, USA). We separated arsenite (As_3_), arsenate (As_5_), MMA, and DMA by high-performance liquid chromatography (Waters 501; Waters Associates, Milford, MA, USA) using a Nucleosil 10u SB 100A column (Phenomenex, Torrance, CA, USA). Individual species using hydride-generated atomic absorption spectrometry (Flow Injection Analysis System 400AA 100; Perkin-Elmer, Waltham, MA, USA) as described by Hsueh et al. (1998). InAs was defined as the sum of As_3_ and As_5_. We calculated the relative proportion of each As species (%InAs, %MMA, and %DMA) by dividing the concentration of each species by the TUA concentration (As_3_ + As_5_ + MMA + DMA). This analytical approach eliminates interference from arsenobetanine and arsenocholine, which are non-toxic organic As species found in seafood.

The average LOD, determined by 115 method blanks run on separate days, for As_3_, As_5_, MMA, and DMA were 0.04 μg/L, 0.06 μg/L, 0.05 μg/L, and 0.06 μg/L, respectively. Quality control procedures included spiked samples, where a known amount of As_3_, As_5_, MMA, and DMA standard reagent was added to one sample within each batch. The average percent recovery for 348 spiked samples for As_3_, As_5_, MMA, and DMA were 98.9 ± 6.5%, 100 ± 6.5%, 99.9 ± 6.4%, and 100.1 ± 6.5%, respectively. Replicates of standard solutions were also analyzed during each laboratory day, and all were ± 5% for each As metabolite. Specifically, the percent difference for As_3_, As_5_, MMA, and DMA were −1.0 ± 3.5%, 0 ± 3.9%, −0.3 ± 3.4%, and −1.3 ± 3.4%. We measured urinary creatinine using the kinetic Jaffe method with a Hitachi 7170S autoanalyzer (Tokyo, Japan). Although at least one UAs was detectable in all of the 2,971 urine samples included in this analysis, 46 (1.6%), 264 (8.8%), and 5 (0.2%) samples were below the LOD for InAs, MMA, and DMA, respectively.

### Statistical analysis

The actual values of all UAs, including those below the LOD, were used in all analyses. All urinary As outcomes were positively skewed and transformed using a base-10 logarithm to achieve a more symmetric distribution.

The data structure was complex, with each subject having up to 18 repeated measures clustered on 3 consecutive days within eight sampling periods. Also, participants were clustered within households. We used hierarchical mixed models (SAS PROC MIXED; SAS Institute Inc., Cary, NC, USA) to assess covariate effects, while accounting for the correlation associated with these clusters by including random effects for subject and household as described by Singer (1998). We explored the inclusion of additional random effects for sampling period but found that the models tended to become unstable, and instead accounted for sampling periods through the inclusion of fixed effect indicators. This modeling approach allowed us to investigate sources of variance by apportioning it into household (variability among 47 households), subject (variability among 195 subjects), and residual variance (unexplained variability within a subject) for each urinary As outcome. In a simpler setting with just one source of clustering, for example, repeated meas urements on subjects, the intraclass correlation coefficient (ICC) would be used to assess reliability and variability of repeated measures over time because the ICC simply corresponds to the ratio of the between-subject variance to the total variance. In our setting, we calculated the percentages of variance attributed to household, between subjects, and within subjects, which are analogous to an ICC with values ranging from 0 to 1. Values near 1 indicate high reliability and low intraindividual variability, whereas values near 0 indicate poor reliability and high intraindividual variability. We re-ran models allowing the variance components to differ according to various factors (e.g., sex, As exposure, smoking status, total water intake, and families that switched tube wells during the study) to examine the contribution of these factors on the observed variance.

To determine how well a single urine sample predicted categorical UAs level (i.e., tertiles), we calculated geometric mean values for each UAs (“true”) and compared them with tertiles constructed from a single day, the average of 2 days within consecutive quarters, the average of 3 days in consecutive quarters, and the average of 3 consecutive days within a quarter (“predicted”). The amount of agreement between the “true” and the “predicted” in the highest tertile (sensitivity) and the lowest two tertiles (specificity) were used to evaluate potential misclassification from different sampling strategies.

We used mixed-effects models to evaluate the association between each urinary As outcome and the following fixed effects: log_10_ creatinine (mg/dL), log_10_ drinking water As (μg/L), sex, age, body mass index (BMI), smoking (currently smokes cigarettes vs. does not currently smoke cigarettes), betel nut (currently chews betel nut vs. does not currently chew betel nut), Ramadan (sample collected during days of fasting vs. sample collected during the rest of the year), season (monsoon months, 1 June through 30 September; warm months, 1 March through 31 May; and cold months, 1 October through the end of February), and day (1, 2, or 3 within sampling period). All continuous variables were centered at their mean. Each model included the nested random-effects variance structure described above. Geometric means for each UAs were provided for the fixed effects in the mixed model to facilitate interpretability.

We evaluated analytical robustness by repeating each analysis and *i*) excluding extreme outliers, including four InAs, four MMA, and two DMA samples that had values more than three standard deviations above or below the mean, and *ii*) excluding observations in the top and bottom 10% of the observed distribution. All analyses were performed using SAS, version 9.1 (SAS Institute Inc.).

## Results

Of the 195 individuals from 47 households, 94% initially provided a urine sample. This participation rate declined to 74% after 2 years. Of the available participants, six never provided a urine sample, although they participated in other aspects of the biomonitoring study. No participant requested to be withdrawn from the study, and samples that were not collected were most likely due to individuals being absent from the home during scheduled collection visits. In addition, 29 urine samples were not analyzed for all UAs. Thus, we included a total of 2,971 urine samples in this analysis, reflecting contributions from 195 participants residing in 47 households. Of these samples, 33%, 27%, and 40% were collected in monsoon, summer months, and winter months, respectively.

Table 1 presents the general characteristics of this study population. Table 2 presents the distributions of drinking water As levels and UAs for each sampling period. We measured at least one UAs in all samples, although we observed considerable variation in UAs with MMA being least prevalent. Overall, 33% of the drinking water samples exceeded the Bangladesh drinking water standard of 50 μg As/L, and the majority of the participants (61.7%) were exposed to As in their drinking water, although approximately one-third of the drinking water samples had no detectable level of As. All participants were informed of the As levels in their tube well, and six households (*n* = 25) installed a new tube well during the course of the study after being told that their current tube well exceeded the Bangladesh drinking water standard.

We estimated the proportions of the observed total variance attributed to household and subject for all UAs (Table 3). When UAs were expressed as a percentage of the TUA, variability within subjects, between subjects, and between households explained 83–84%, 12–15%, and 1–3% of the observed total variance, respectively. We defined generalization of the ICCs as the sum of the between-household and between-subject variances, divided by the total variance. These ICCs were poor (%InAs, 0.16; %MMA, 0.16; %DMA, 0.17), indicating that the percentage of each urinary As species was not stable within an individual over the 2-year period. However, when UAs were expressed as concentrations rather than percentages, variability within subjects, between subjects, and between households explained 51–65%, 9–11%, and 26–40% of the observed total variance, respectively. The reduction of within-subject variability associated with using the concentration of each UAs increased ICCs to 0.41, 0.35, 0.47, and 0.49 for InAs, MMA, DMA, and TUA. This indicated that the concentration of each UAs was moderately stable within the individual over the 2-year observation period. Excluding the 25 individuals who installed new tube wells, removing extreme outliers, or restricting the analysis to the 10th to 90th percentile of the UAs distribution did not change these results.

The proportion of variance models were stratified by sex, smoking, and exposure to As-contaminated water to examine how the variability in UAs differed among these categories (Table 4). For example, males and females exhibited similar inter- and intraindividual variability in all urinary outcomes except at the household level, where household affiliation explained more of the total observed variability for males. The effect of smoking on the variability of UAs was examined only in men because no woman reported smoking in this population. Males who reported smoking at least 10 cigarettes per week explained little to no observed interindividual variability in methylated UAs MMA (0–2%) and DMA (0–6%) but slightly increased the intra-individual variability in all UAs compared with individuals who did not report smoking. Exposure to As-contaminated water (expressed as tertiles) was also associated with increased variability with increased tertile of exposure, but the intraindividual variance was always greatest in the lowest exposure tertile.

The effect of several factors on mean UAs were examined using multivariate linear mixed-effects models (Table 5). For continuous variables, we observed a positive significant association between As-contaminated drinking water and higher InAs, MMA, DMA, and TUA. Increased creatinine concentrations had the largest effect on all UAs, with increasing creatinine concentrations associated with increasing InAs, MMA, %MMA, DMA, %DMA, and TUA, although increasing creatinine concentrations were inversely associated with %InAs. Increasing age in years was associated with decreased MMA but increased DMA. BMI was included in the models as a quadratic term, which displayed an inverted U-shaped relationship with MMA, with increasing BMI associated with increased MMA and body mass squared associated with decreased MMA.

We also examined several categorical variables for their effect on mean UAs. We observed that, on average, males had higher MMA but lower DMA and TUA compared with females. Individuals who reported chewing betel nuts had higher concentrations of MMA and %MMA compared with individuals who did not report chewing betel nuts. Samples collected during Ramadan (a month of fasting during daylight hours) had higher urinary As levels for all outcomes compared with samples collected at other times of the year. Also, individuals who reported that they smoked had lower concentrations of DMA compared with nonsmokers. When the effect of smoking was examined in males only, smoking was associated with higher InAs compared with not smoking, although this association did not reach statistical significance. However, when we expressed As metabolites as a percentage of TUA, the %InAs was higher and %DMA was lower in males who reported smoking compared with males who did not report smoking.

The season in which the sample was collected also influenced all UAs. Compared with samples collected in the monsoon months (June–September), samples collected in cooler months (October–February) were associated with the lower InAs, MMA, DMA, and TUA. Samples collected in the warmer months (March–May) also had lower InAs, MMA, DMA, and TUA compared with samples collected in the monsoon months. This resulted in lower %InAs and %MMA but higher %DMA during cooler months, but we observed an opposite effect during warmer months. We also observed day-to-day differences in mean UAs. Compared with day 3, InAs was higher on day 1 and day 2. DMA concentrations were highest on day 1 and decreased on day 2, although this trend did not reach statistical significance. TUA also showed a similar trend with the highest TUA concentrations on day 1 and lower concentrations on day 2 compared with day 3.

Table 6 presents the sensitivity and specificity of different sampling strategies. The proportion of participants that truly had the highest 2-year average UAs levels (top 33%) that would be identified as such using a single urine sample anytime throughout the 2-year observation period (i.e., sensitivity) was 0.76, 0.76, 0.84, and 0.85 for InAs, MMA, DMA, and TUA, respectively. The proportions of participants that truly had the lowest UAs levels (lower 66%) that were classified correctly (i.e., specificity) were 0.74, 0.78, 0.79, and 0.79 for InAs, MMA, DMA, and TUA, respectively. This indicated that a single urine sample adequately classified an individual’s urinary As profile, although both sensitivity and specificity improved with multiple sample collection. Because TUA is commonly used as a biomarker of exposure, we examined how well it predicted high (top 33%) and low (bottom 66%) tube well As concentrations. Although highly specific, TUA was only moderately sensitive at accurately characterizing drinking water exposures. Removing the 25 participants from the six households that installed a new tube well during the course of the study did not substantially influence the sensitivity or specificity estimates (data not shown).

## Discussion

Although we observed that urinary As concentrations were moderately reproducible within this population over a 2-year period, the percentage of individual UAs that are used to evaluate As methylation capacity were not. This differs somewhat from the conclusions of Concha et al. (2002) and Steinmaus et al. (2005), who both reported that the percentage of UAs were relatively stable within individuals over a 5-day and 258-day interval. For instance, Steinmaus et al. (2005) reported ICCs of 0.45, 0.46, and 0.49 for %InAs, %MMA, and %DMA, respectively. Although these ICCs are similar to what we reported for the concentration of UAs, they are much higher than what we observed for the percentage of UAs. However, the different results of these studies could be explained by different environmental and behavioral factors unique to each population. For instance, in Bangladesh, participants rely exclusively on local water sources for all their drinking water. This differs from the United States, where more beverage options are available. In addition, As exposures in the United States may be more constant than in Bangladesh, where As mitigation programs encourage families to avoid As-contaminated water by using water filters or sharing a neighbor’s tube well that is considered safe (Hanchett et al. 2002). Also, dietary As exposures may be more significant and more variable in Bangladesh than in the United States, particularly for individuals with low tube well As exposures (Kile et al. 2007; Smith et al. 2006). Also, As metabolism may be sensitive to time-varying events that were not captured in our study but play a more important role in Bangladesh, such as folate intake.

It is also likely that the low intraindividual correlations with respect to %InAs, %MMA, and %DMA were a function of the inherent instability of ratios with small denominators as seen in approximately half of this population who had low As exposure. For these individuals, other sources of As exposure such as diet could be an important contributor to the observed variability. We found evidence supporting this conclusion when we stratified the observed variability by tube well As exposure. Specifically, individuals in the lowest tertile of exposure had the greatest intraindividual variability compared with higher tertiles, yet the magnitude of the estimates for the within-subject variance were similar across exposure tertiles. This indicated that the variability associated with the individual was relatively constant and independent of tube well As exposure. Other nondrinking water sources of As exposure would also explain the low specificity of TUA for correctly identifying individuals with low tube well As exposure.

We further examined variance in UAs by stratifying models on known characteristics that have been suggested to influence UAs. Parsing the variance in this fashion demonstrated that unknown factors within subjects remained the largest source of variance for all UAs, although some interesting patterns did emerge. For instance, compared with females, males had less intraindividual and less inter-individual variability but more between-household variability with the strongest effect observed for MMA. This between-household difference could be a function of genetic factors because traditional Bangladeshi households are organized along paternal bloodlines where males remain in the household after marriage. Thus, males from the same household were more highly related (e.g., offspring or sibling) compared with females, who were either the maternal blood relative or an unrelated spouse. It was also interesting to note that smoking explained a portion of the inter-individual variability in InAs but little to none of the interindividual variability in methylated As metabolites MMA and DMA. This suggested that smoking interfered with As methylation capacity. We also examined the variance stratified by season (data not shown). Although the proportion of variance between individuals varied by season, we observed no difference at the household level, which suggested that unknown behavioral or dietary differences that varied with season contributed to the observed interindividual variance.

The results from the mixed-effects models on the average UAs concentrations showed that sex, tube well As exposure, Ramadan, age, BMI, smoking, chewing betel nuts, urinary creatinine, season, and day influenced mean UAs. Many of these effects have been reported in other studies. For instance, several studies show that males excrete more InAs and less DMA compared with females and that DMA excretion increases with age (Hopenhayn-Rich et al. 1996; Vahter 1999). Thus, it would seem that urinary As concentrations and the percentage of UAs have different utilities as biomarkers with concentrations reflecting exposure and percentages reflecting susceptibility. Through careful examination of the differences between urinary As concentrations and the percentage of UAs, it is possible to distinguish between associations that could be driven by exposure and associations driven by biological responses. For instance, the concentrations of all UAs increased with tube well As levels, but only the percentage of InAs and MMA—and not DMA—increased with As exposure. This suggested the possibility that As metabolism was slowed or possibly saturated as As exposure increased. In the case of smoking, we found no significant association with urinary As concentrations, but individuals who reported smoking excreted a higher percentage of InAs and a lower percentage of DMA compared with individuals who did not report smoking. This suggested that smoking interfered with As methylation but was not necessarily a source of As exposure. Because the percentages of UAs were sensitive to changes in both the numerator and denominator, it was useful to examine how factors influenced both the relative percentage and the concentration of urinary As to gain greater insight into As metabolism.

In conclusion, this is the longest prospective biomonitoring study of As exposure published to date. We observed that urinary As ratios were poorly reproducible within the individual over a 2-year observation period but that urinary As concentrations were fairly reproducible. Because intraindividual variability can contribute to misclassification, using urinary As concentrations would reduce this source of measurement error and potentially improve statistical precision. Also, by reporting both urinary As concentrations and the percentage of UAs, it was possible to examine how risk factors influenced As methylation. Finally, unknown time-varying factors appeared to be the largest contributor to the observed inter- and intraindividual variability in As metabolism. Considering that an individual’s ability to metabolize As appears to influence susceptibility to chronic As exposure, more research is needed to identify those behavioral and environmental factors that influence As metabolism.

## Figures and Tables

**Table 1:** Population characteristics of 195 adults.

Characteristic	Percent	Mean ± SD	Range
Age (years)		33.4 ± 13	15–77
BMI		20.6 ± 3.5	13–30
Male	42.3		
Current smoker	18.3		
Chews betel nut	26.3		

**Table 2:** The distribution of UAs, the percent of each urinary As species, and tube well As concentrations among 195 participants.

			Percentile						
Parameter	GM	Minimum	10th	25th	50th	75th	90th	95th	Maximum
TW As (μg/L)	8.8	ND	0.5	0.7	12.5	96.7	160.4	327.0	567.7
TUA (μg/L)	32.3	4.3	13.9	20.6	30.2	45.9	72.9	135.6	293.0
InAs (μg/L)	3.0	0.8	1.6	2.2	3.8	6.0	10.6	17.4	51.1
Percent InAs	11.1	5.5	8.5	10.5	12.6	15.2	18.0	19.8	35.7
MMA (μg/L)	2.1	ND	1.1	1.6	2.6	4.6	8.0	15.1	50.9
Percent MMA	7.4	ND	5.0	6.5	8.5	11.3	14.0	16.6	24.4
DMA (μg/L)	20.4	3.3	10.3	16.3	22.8	36.7	54.2	103.1	200.1
Percent DMA	76.6	5.3	68.8	74.4	78.8	81.6	85.0	86.0	88.4

Abbreviations: GM, geometric mean; ND, samples less than the LOD; TW, tube well

**Table 3:** The proportion of the observed variance attributed to between households, between subjects, and within subjects for all 195 participants, including and excluding the 25 participants who had a new tube well installed during the 2-year observation period.

	All observations (*n* = 195)			No. switched wells (*n* = 170)		
	Estimate^a^	Percent of total	ICC	Estimate^a^	Percent of total	ICC
Log TUA (μg/L)						
Between household	68.9	40	—	71.2	41	—
Between subject	16.6	10	—	15.9	9	—
Within subject	87.9	51	0.49	86.8	50	0.50
Log InAs (μg/L)						
Between household	76.5	30	—	78.2	31	—
Between subject	28.9	11	—	26.9	11	—
Within subject	150.0	59	0.41	149.5	59	0.41
Log %InAs						
Between household	0.9	1	—	0.3	0	—
Between subject	12.9	15	—	13.5	15	—
Within subject	74.0	84	0.16	75.1	84	0.16
Log MMA (μg/L)						
Between household	94.9	26	—	99.8	28	—
Between subject	30.8	9	—	28.1	8	—
Within subject	234.2	65	0.35	224.4	64	0.36
Log %MMA						
Between household	7.8	5	—	7.8	5	—
Between subject	18.5	12	—	18.1	12	—
Within subject	133.2	84	0.16	126.2	83	0.17
Log DMA (μg/L)						
Between household	65.2	37	—	67.1	38	—
Between subject	17.1	10	—	16.8	10	—
Within subject	93.9	53	0.47	92.4	52	0.48
Log %DMA						
Between household	0.2	3	—	0.1	2	—
Between subject	1.0	13	—	0.9	13	—
Within subject	5.9	83	0.17	5.8	85	0.15

Variance estimates were multipiled by 1,000 to aid interpretability.

**Table 4:** Percentage of total variance attributed to between-household, between-subject, and within-subject variance, estimated by stratifying models by sex, smoking (yes = currently smokes, no = never/former smoker), and tube well As exposure tertiles.

	Sex		Smoking		Tube well As (μg/L)		
	Female	Male	Yes	No	Low	Medium	High
Log total As (μg/L)							
Between household	34.7	41.2	40.8	38.5	9.8	31.1	37.9
Between subject	13.1	9.6	8.3	17.9	13.5	15.6	9.4
Within subject	52.2	49.2	50.9	43.6	76.6	53.3	52.7
Log InAs (μg/L)							
Between household	27.1	34.4	26.8	37.2	6.1	26.2	30.9
Between subject	10.9	9.5	12.2	13.5	16.2	13.8	9.7
Within subject	62.0	56.1	61.1	49.4	77.6	60.0	59.4
Log %InAs							
Between household	1.6	4.6	0.0	13.1	1.7	0.6	3.7
Between subject	10.7	13.5	11.4	7.3	11.9	17.0	17.2
Within subject	87.7	82.0	88.6	79.6	86.3	82.4	79.1
Log MMA (μg/L)							
Between household	19.2	34.4	38.8	34.5	4.4	7.5	16.2
Between subject	9.1	5.8	1.8	10.1	10.1	6.5	9.4
Within subject	71.7	59.8	59.4	55.4	85.6	86.0	74.4
Log %MMA							
Between household	2.1	11.5	18.1	9.5	4.2	3.7	8.1
Between subject	7.8	7.4	0.0	10.3	6.2	13.0	24.5
Within subject	90.1	81.2	81.9	80.3	89.7	83.3	67.4
Log DMA (μg/L)							
Between household	32.6	37.7	37.0	35.8	8.3	23.1	10.8
Between subject	13.9	9.3	5.9	18.7	12.8	10.3	10.2
Within subject	53.5	53.0	57.1	45.5	78.9	66.7	79.0
Log %DMA							
Between household	0.6	6.9	8.7	3.6	3.1	0.3	4.7
Between subject	7.9	12.4	0.0	28.7	16.7	15.2	13.8
Within subject	91.5	80.6	91.3	67.7	80.3	84.5	81.5

Models examining smoking were restricted to males only.

**Table 5:** Geometric means for all urinary As outcomes stratified by sex, tube well (TW) As tertiles, Ramadan, age, BMI, BMI^2^, smoking status, betel nut use, average creatinine, season, and day.

	InAs					MMA					DMA					TUA		
Variable	No.	GM (μg/L)	*p*-Value	GM (%)	*p*-Value	No.	GM (μg/L)	*p*-Value	GM (%)	*p*-Value	No.	GM (μg/L)	*p*-Value	GM (%)	*p*-Value	No.	GM (μg/L)	*p*-Value
Overall	2,914	3.0	—	10.9	—	2,739	2.2	—	7.4	—	2,936	20.9	—	76.9	—	2,939	27.7	—
Sex																		
Male	1,234	3.5		12.0		1,178	2.9		9.3		1,239	21.6		73.9		1,239	29.2	
Female	1,680	2.7	0.9	10.2	0.006	1,561	1.7	0.04	6.2	< 0.001	1,697	20.4	0.001	79.1	0.001	1,700	25.7	0.01
TW As (μg/L)																		
< 1	1,111	1.8		10.6		1,022	1.3		7.0		1,124	13.1		77.8		1,126	16.7	
1–50	846	3.0		10.7		793	2.1		7.0		852	21.4		78.2		852	27.4	
> 50	957	5.5	< 0.001	11.5	0.03	924	4.1	< 0.001	8.3	0.87	960	35.4	< 0.001	74.6	0.007	961	47.2	< 0.001
Ramadan																		
Yes	289	4.0		10.3		273	2.7		6.6		289	30.2		78.3		289	38.5	
No	2,625	2.9	0.008	11.0	0.93	2,466	2.1	0.001	7.5	0.03	2,647	20.1	0.001	76.7	0.92	2,650	26.1	0.004
Age (years)																		
15–25	1,058	3.4		12.1		992	2.2		7.3		1,064	21.1		75.7		1,065	27.7	
26–35	788	3.3		11.4		750	2.1		6.8		794	22.2		77.6		794	28.6	
36–45	602	2.5		10.2		559	2.1		7.9		604	19.4		77.0		606	24.9	
≥ 46	466	2.3	< 0.002	8.7	< 0.001	438	2.3	0.94	8.0	0.46	474	20.5	0.01	78.3	0.001	474	26.2	0.11
BMI^a^																		
< 18	520	2.8		11.1		496	2.3		8.7		525	19.2		75.8		526	25.2	
18–25	1,966	3.1		11.0		1,845	2.2		7.2		1,979	21.1		76.7		1,987	27.4	
> 25	428	2.9	0.05	10.1	0.36	398	2.0	0.34	6.6	0.99	432	22.2	0.14	78.9	0.40	432	28.1	0.08
Smoker^b^																		
Yes	532	4.0		13.5		508	3.1		9.8		532	21.3		71.3		532	21.3	
No	2,382	2.8	0.13	10.4	< 0.001	2,231	2.0	0.49	6.9	0.11	2,404	20.8	0.02	78.1	0.004	2,407	26.5	0.15
Betel nut^b^																		
Yes	763	2.7		9.9		722	2.5		8.5		773	20.6		77.2		775	26.4	
No	2,151	3.1	0.48	11.3	0.24	2,017	2.1	0.01	7.0	0.001	2,163	21.0	0.31	76.7	0.10	2,164	27.3	0.48
Log creatinine (mg/dL)																		
Low	954	1.6		11.1		845	1.1		7.1		971	10.6		75.7		974	13.9	
Medium	994	3.1		10.8		944	2.2		7.4		999	21.8		77.2		999	28.2	
High	966	5.5	< 0.001	10.8	< 0.001	950	4.0	< 0.001	7.7	< 0.001	966	39.6	< 0.001	77.7	< 0.001	966	51.0	< 0.001
Season^b^																		
Winter	1,120	2.3	< 0.001	10.0	0.13	1,027	1.5	< 0.001	5.8	< 0.001	1,130	18.1	< 0.001	79.5	0.007	1,132	22.6	< 0.001
Summer	819	3.2	0.09	2.2	0.001	775	2.5	0.04	8.9	0.14	824	19.5	< 0.001	73.9	0.003	825	26.3	< 0.001
Monsoon	975	3.8	—	10.9	—	937	3.0	—	8.3	—	982	26.1	—	76.4	—	982	34.2	—
Day^b^																		
1^c^	1,356	3.3	< 0.001	11.4	< 0.001	1,260	2.3	0.94	7.3	0.17	1,371	22.0	0.09	76.9	0.40	1,372	28.5	0.03
2	800	2.8	0.10	10.9	0.01	758	2.0	0.18	7.3	0.28	805	19.4	0.06	76.1	0.02	805	25.5	0.46
3	758	2.7	—	10.0	—	721	2.2	—	7.6	—	760	20.6	—	77.6	—	762	26.3	—

GM, geometric mean. *p*-Values are from the test for fixed effects using log-transformed data in mixed models with nested random effects for family, subject, and quarter.

a BMI modeled as quadratic BMI^2^ (*p*-values: %InAs, 0.34; InAs, 0.05; %MMA, 0.86; MMA, 0.27; %DMA, 0.37; DMA, 0.14; TUA, 0.08).

b Test of two groups (males vs. females; Ramadan vs. not Ramadan; smoker vs. nonsmoker; betel nut vs. not betel nut; winter vs. monsoon; summer vs. monsoon; day 1 vs. day 3; day 2 vs. day 3).

c For sampling periods 1–3, samples were collected only on day 1; for sampling periods 4–8, samples collected on 3 consecutive days.

**Table 6:** Sensitivity for predicting participants with highest overall mean InAs, MMA, DMA, TUA (top 33%) and specificity for predicting participants with lowest (bottom 66%) overall mean InAs, MMA, DMA, and TUA.

	Log InAs		Log MMA		Log DMA		Log TUA		Log TUA to TW	
Sampling scenario	Sensitivity	Specificity	Sensitivity	Specificity	Sensitivity	Specificity	Sensitivity	Specificity	Sensitivity	Specificity
One sample	0.76	0.74	0.76	0.78	0.84	0.79	0.85	0.79	0.81	0.56
Two samples (3 months apart)	0.83	0.86	0.81	0.78	0.90	0.79	0.90	0.86	0.84	0.54
Three samples (each 3 months apart)	0.86	0.89	0.88	0.82	0.91	0.87	0.91	0.85	0.83	0.51
Three samples (3 consecutive days)	0.83	0.86	0.82	0.78	0.91	0.76	0.90	0.81	0.87	0.57

TW, log_10_ tube well As (μg/L). We also examined TUA sensitivity and specificty for predicting highest (top 33%) and lowest (bottom 66%) overall drinking water As exposure.
